# Supplementary material for: Identification of recurrent dynamics in distributed neural populations
Source: PLoS Comput Biol. 2025 Feb 6;21(2):e1012816. doi: 10.1371/journal.pcbi.1012816 (PMC11838891; doi:10.1371/journal.pcbi.1012816)
Supplement: Attachment — Submitted filename: PLOS_CBIO_review.pdf [file pcbi.1012816.s001.pdf]

Summary:

In this paper, the authors explore the use of time-varying autoregression with low-rank tensors (TVART) to identify recurrent linear dynamics in simulated neural population data. The paper tests TVART on stochastic neural mass models with multiple stable attractors, including simple two-population models and more complex models based on human brain connectivity. The authors demonstrate that TVART can recover the underlying attractor structure through the clustering of temporal modes and examine how prediction time delay impacts the ability to separate different dynamical regimes.

This paper's detailed investigation of TVART, its properties, and its effectiveness in recovering meaningful dynamical structures is interesting and significant. However, the paper could be improved in a few areas, as described below.

Major comments and questions:

- Clarity
  - Some key concepts or terms should be defined when they are first used in the text. For example, the window size M, prediction delay, and temporal delay prediction are important terms that are used in the text before it is defined. In addition, although it is defined in the Methods section what the temporal modes of TVART are, it would be helpful for the readers if the paper explains in words or visually in the main text what these temporal modes are.
  - The paper needs more details on how the model fitting works for TVART and how the users should interpret the fitted model. For example, it is unclear how we can extract information about the attractors from the fitted TVART (Lines 147-148).
- Experimental comparisons with competing methods
  - The current experiments with TVART are interesting and promising, but it should be compared with competing methods. Without proper comparisons, we cannot conclude whether TVART is doing well enough in identifying recurrent dynamics in neural data. For comparisons, the authors should consider switching models such as switching linear dynamical systems, autoregressive hidden Markov models, and related variants [1; Ch. 29]. Such models have the advantage of bypassing the additional clustering step of TVART, and the number of discrete states for these switching models can be determined via cross-validation. I am curious whether such unsupervised methods can perform as well as TVART.
- Related work
  - The paper should cite recent low-rank tensor-based methods [2-4] and compare and contrast TVART with them. The authors should be clear in what strengths or weaknesses TVART has compared to these similar methods.
- Figure interpretability
  - I had trouble understanding panels E and F of Figures 3 and 4. Are the red dots disjoint sets across clusters? Additional details would be helpful for the readers.

- I enjoyed the level of details in Section "Derivation of MSE around a single stable fixed point".
- Hyperparameter selection and effect of initialization
  - Lines 486-492 list the hyperparameters used for TVART. How are these hyperparameters selected? A brief discussion of how the TVART hyperparameter selection process would be beneficial.
  - To my understanding, CP-decomposition is prone to local minima. Since TVART imposes a rank constraint via rank R CP-decomposition, I am curious whether TVART also suffers from local minima and how different initializations affect the model fits.

Minor comments:

- I would use a different word (e.g., "demonstrating") instead of "proving" in line 53.
- The paper is missing the word "Figure" in lines 150 and 152.
- I may have missed it, but is there a plot that demonstrates that the results are robust to the "choice of regularization method" (Line 217)? Do different colors in Figure 5 panels C and D correspond to different regularization strengths?
- For panels C and D of Figures 3 and 4, I would try to decrease the size of the dots a bit.

Reference:

[1] Murphy, Kevin P. (2023). Probabilistic Machine Learning: Advanced Topics. MIT Press. URL: http://probml.github.io/book2

[2] Zhang, W., Cribben, I., & Guindani, M. (2021). Bayesian time-varying tensor vector autoregressive models for dynamic effective connectivity. *arXiv preprint arXiv:2106.14083*.

[3] Wang, D., Zheng, Y., Lian, H., & Li, G. (2022). High-dimensional vector autoregressive time series modeling via tensor decomposition. *Journal of the American Statistical Association*, *117*(539), 1338-1356.

[4] Lee, H. D., Warrington, A., Glaser, J., & Linderman, S. (2023). Switching autoregressive low-rank tensor models. *Advances in Neural Information Processing Systems*, *36*, 57976-58010.
