# Supplementary material for: Identification of recurrent dynamics in distributed neural populations
Source: PLoS Comput Biol. 2025 Feb 6;21(2):e1012816. doi: 10.1371/journal.pcbi.1012816 (PMC11838891; doi:10.1371/journal.pcbi.1012816)
Supplement: Attachment — Submitted filename: Response to Reviewers.pdf [file pcbi.1012816.s003.pdf]

We greatly appreciate the reviewers' recognition of our modifications and their additional feedback. We thank the reviewers for their time and effort in helping us further improve our manuscript. In response to the latest comments, we have revised and expanded our manuscript accordingly. Please see our itemized responses below:

Reviewer #1

Prediction farther into the future (termed look-ahead in prior works) has been studied in multiple models, including in LDS, SLDS, and dLDS models similar to TVART, as well as more general nonlinear systems (RNNs).

We are thankful for the reviewer bringing these works to our attention. We have expanded the discussion section to better connect our study to this broader context. We reproduce the relevant lines (440-463 in the revised manuscript) below for your convenience:

"Alternatively, autoregression can be performed based on multistep prediction error. In general, autoregressive methods, including TVART, can easily accommodate methods that include multiple time lags, although this increases the number of parameters and makes the classification problem harder. Iterating a solution over multiple steps can also be used as a strategy for predicting over longer time horizons; such an approach avoids increasing the number of parameters (Marcellino, 2006). Nonetheless, the iteration of a linear solution may not be optimal if the underlying dynamics are non-linear. This challenge may be addressed by a novel training method named LINOCS (Lookahead Inference of Networked Operators for Continuous Stability) developed by Mudrik et al (Mudrik, 2024b). LINOCS utilizes multiple lookahead estimations to improve the stability and accuracy of dynamical system inference. Future work could explore the combination of LINOCS and TVART in the recovery of attractor dynamics in multistable neural systems.

Moreover, inference by looking further into the future has been shown to be more broadly beneficial. In the case of chaotic neural dynamics, time delay embedding and piecewise linear recurrent neural networks have been leveraged for faithful reconstruction of dynamics (Hess, 2023). Such an approach in combination with generalized teacher forcing was leveraged by Hess et al (Hess, 2023) for learning chaotic dynamics. Similarly, RNN-transducers have been shown to benefit from using lookahead tokens, for instance by reducing error in speech to text conversion tasks (Unni, 2023). These studies along with the results presented here underscore the importance of considering the forecast horizon when inferring interpretable neural dynamics. A combination of approaches will probably be required to deal with the full complexity of full brain dynamics, which may include the

existence of multiple chaotic attractors (Terada, 2024) as well as a plethora of external stimuli.”

The relevant references added are (in order cited):

(Marcellino, 2006): Massimiliano Marcellino, James H Stock, and Mark W Watson. A comparison of direct and iterated multistep ar methods for forecasting macroeconomic time series. 2006. Journal of econometrics, 135(1-2): 499–526.

(Mudrik, 2024b): Mudrik, N., Yezerets, E., Chen, Y., Rozell, C. and Charles, A., 2024. LINOCS: Lookahead Inference of Networked Operators for Continuous Stability. Transactions of Machine Learning Research 2024.

(Hess, 2023): Hess, F., Monfared, Z., Brenner, M. and Durstewitz, D., 2023, July. Generalized Teacher Forcing for Learning Chaotic Dynamics. International Conference on Machine Learning (pp. 13017-13049). PMLR.

(Unni, 2023): Vinit S Unni, Ashish Mittal, Preethi Jyothi, and Sunita Sarawagi. Improving rnn-transducers with acoustic lookahead. 2023. arXiv preprint arXiv:2307.05006.

I also regret that I was not precise about the log-scale for Panels G and H: I meant the y-axis should be on a log scale, not the x-axis. This will emphasize the location of the peak by highlighting subtle height differences into the upper range of values.

We appreciate the clarification. We have amended Figures 3 to 6, and they now have both axes on a log scale. We decided to keep the log scale in the x-axis in this latest version, since in our opinion it improved the readability of the figures. Please see the amended figures below (note that in some cases the changes in the y axis due to the log scale are subtle due to the small range of values in the y axis).

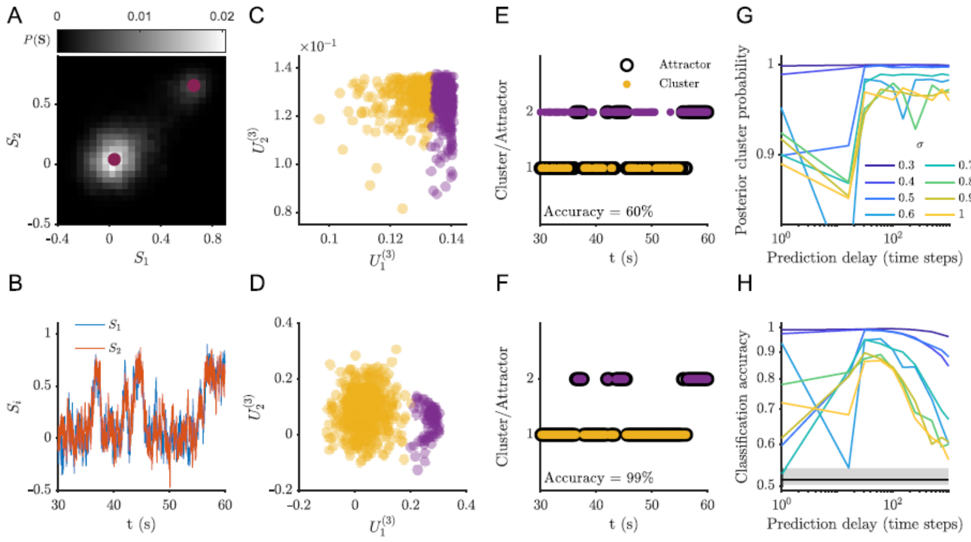

Fig 3. TVART can recover attractor dynamics with high accuracy for a two attractor network. (A) Sample simulation ($\sigma = 0.5$) phase space probability distribution. (B) Sample of simulation time series. (C) Clustering of the temporal modes for one step prediction regression and (D) for 31 steps ahead prediction regression. (E) Comparison of the attractor and cluster indices for one step prediction regression and (F) for 31 steps ahead prediction regression, the classification accuracy for (E) was 59.7% and for (F) 99.0%. (G) Mean posterior cluster probability as a function of prediction delay for several noise levels. (H) Classification accuracy as a function of prediction delay for several noise levels, median chance level is indicated by the black horizontal line and the shaded region encompasses the 5th and 95th percentiles.

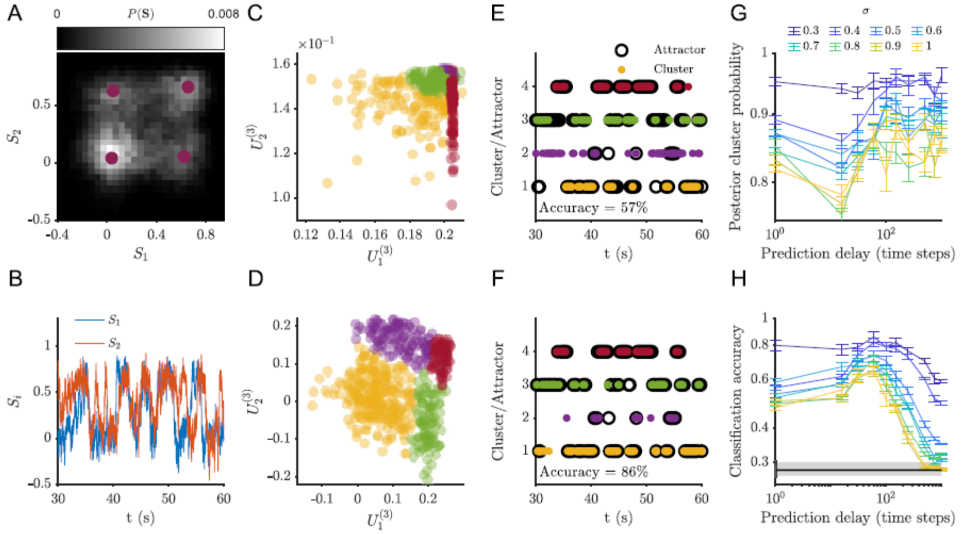

Fig 4. There is an optimal prediction delay for identification of attractor dynamics.(A) Sample simulation ($\sigma = 0.5$) phase space probability distribution. (B) Sample simulation time series (just a subset is depicted for clarity). (C) Clustering of the temporal modes for one step prediction regression and (D) for 31 steps ahead prediction regression. (E) Comparison of the attractor and cluster indices for one step prediction regression and (F) for 31 steps ahead prediction regression, the classification accuracy for (E) was 56.8% and for (F) 86.1%. Mean posterior cluster (G) Mean posterior cluster probability as a function of prediction delay for several noise levels. (H) Classification accuracy as a function of prediction delay for several noise levels. For (G) and (H) results are averaged over 10 TVART fits and error bars represent the standard error of the mean).median chance level is indicated by the black horizontal line and the shaded region encompasses the 5th and 95th percentiles.

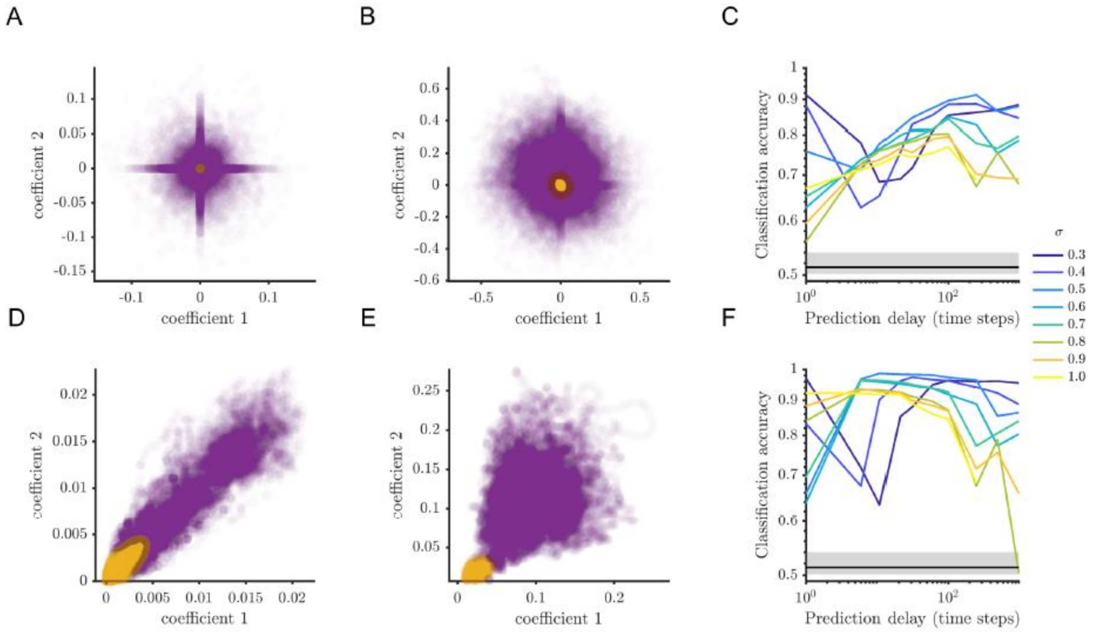

Fig 5. dLDS attractor recovery also benefits from increasing prediction delay dLDS coefficients for the sample simulation with ($\sigma = 0.5$). (A) Coefficients for 1-step delay fits and (B) for 31-step delay fits. (C) Classification accuracy based on dLDS coefficients as a function of prediction delay. (D) dLDS coefficients after taking absolute value and smoothing by a centered boxcar filter over 100 steps for 1-step delay fits and for (E) 31-step delay fits. (F) Classification accuracy based on smoothed absolute value dLDS coefficients as a function of prediction delay. Transforming the dLDS coefficients substantially improved attractor identification. The classification accuracy corresponding to the scatter plots are: (A) 71.4%, (B) 81.7%, (D) 58.1%, and (E) 98.2%.

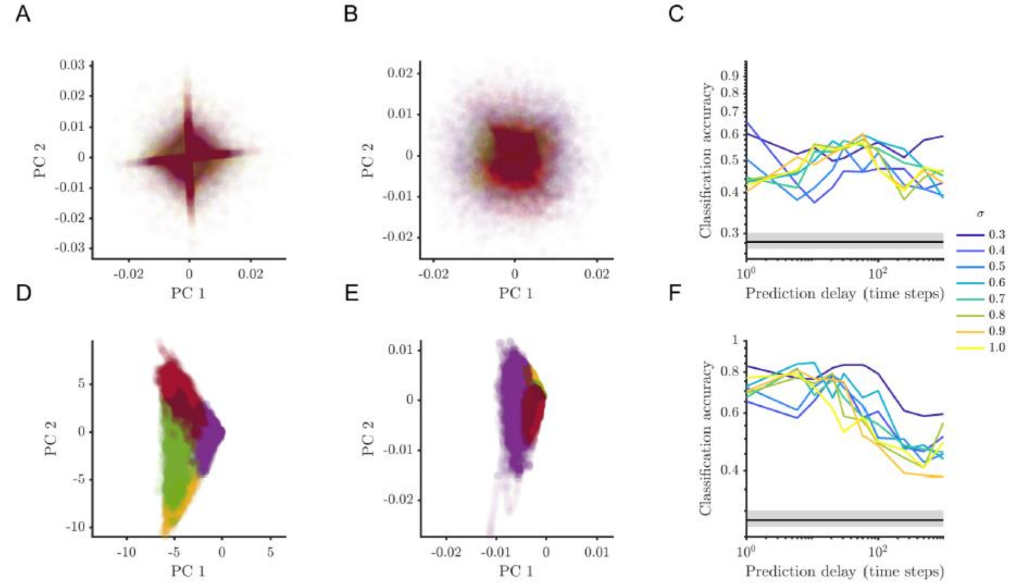

Fig 6. dLDS attractor recovery benefits from temporal smoothing dLDS coefficients for the sample simulation with ($\sigma = 0.5$). (A) Principal components of coefficients for 1-step delay fits and (B) for 31-step delay fits. (C) Classification accuracy based on dLDS coefficients as a function of prediction delay. (D) Principal components of dLDS coefficients after taking absolute value and smoothing over 100 steps for 1-step delay fits and for (E) 31-step delay fits. (F) Classification accuracy based on smoothed absolute value dLDS coefficients as a function of prediction delay. Transforming the dLDS coefficients substantially improved attractor identification. The classification accuracy corresponding to the scatter plots are: (A) 47.9%, (B) 40.7%, (D) 68.6%, and (E) 76.8%.

Reviewer #2

The authors have satisfactorily replied to all my comments, many thanks for your work.

We are pleased to have provided a response that satisfactorily addresses this reviewer's comments.

Reviewer #3

Thank you for the thoughtful revisions to the paper in response to the comments provided. In particular,

• I appreciate the additional discussion of the TVART model and key terminology at the beginning of the relevant subsection. This clarification enhances the paper significantly. However, I believe that lines 166-171 could benefit from further editing to improve sentence clarity.

We have rewritten lines 166-171. They now appear as follows:

"We can identify switching dynamics by examining the temporal modes, $u_k^{(3)}$. Specifically, we look for clusters of TVART temporal modes, which indicate temporal windows with similar linear dynamics. Such similar linear dynamics may occur within segments of the time series that exist within the same attractor. Therefore, TVART can facilitate the identification of attractor dynamics."

• The discussion of hyperparameters and their selection is very helpful. However, I believe there is still an opportunity to address the sensitivity of TVART to hyperparameter selection in more detail. For example, it would be beneficial if the authors could elaborate on the meaning of "less clear clustering" when beta is increased. A quantitative assessment of clustering quality (and conclusions thereof) as a function of beta (and other hyperparameters) would be helpful.

We have expanded the methods section to include a sensitivity analysis of TVART to hyperparameter selection (lines 600-651). We focused on the simulations shown in figures three and four and summarized the results of this new analysis in figures eleven and twelve (see below). Although we spanned four orders of magnitude in the hyperparameters, the values of the metrics we used to quantify sensitivity varied only slightly.

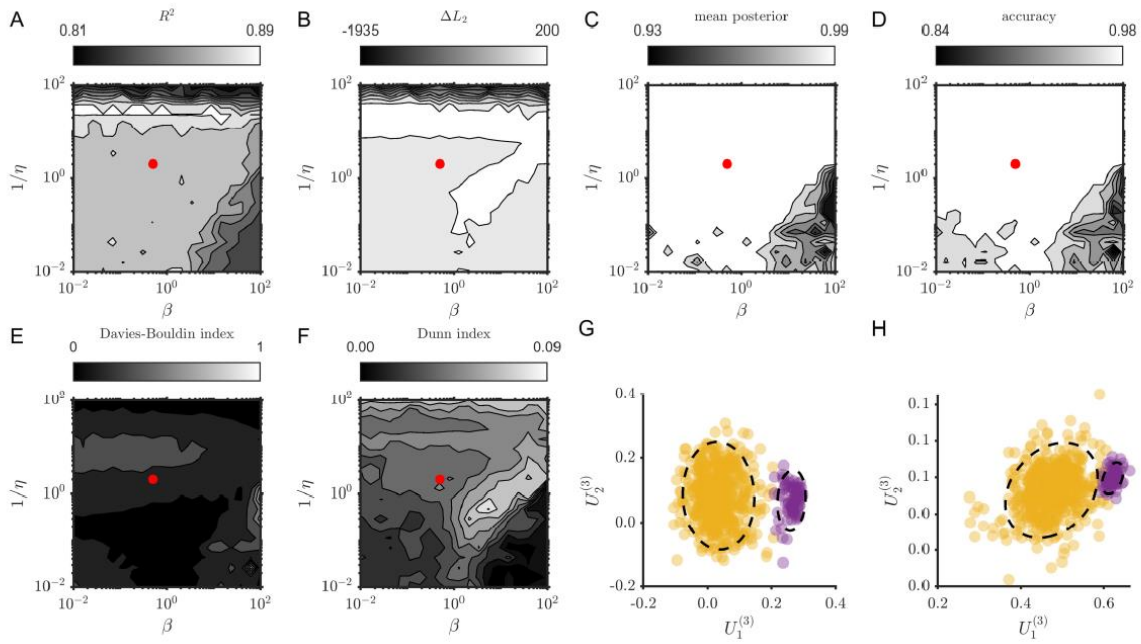

Fig 11. TVART results are robust over a broad range of hyperparameter values. (E-F) Contours of the explored metrics as functions of hyperparameters η and β (increasing $1/\eta$ or β leads to stronger regularization), for two-attractor dynamics. The red dot indicate the values used for the main analysis. (G) Clustering example for $\eta = \beta = 0.48$. (H) Clustering example for $\eta = \beta = 100$. The dashed ellipses are the two sigma contours of the Gaussian components.

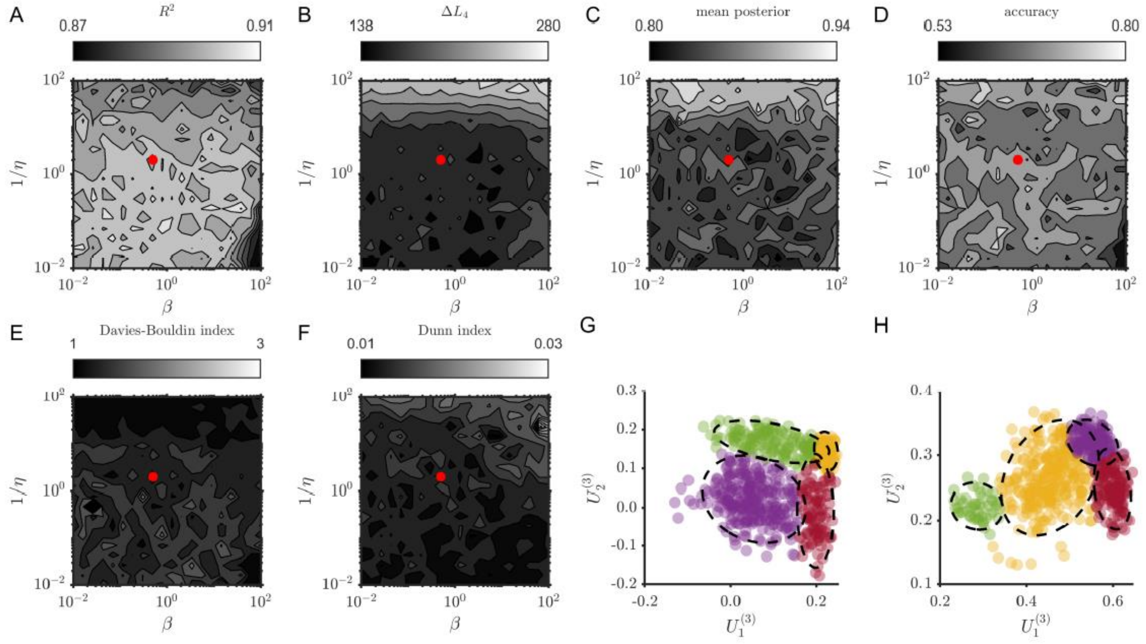

Fig 12. Greater dynamic complexity only slightly increases hyperparameter sensitivity. (E-F) Contours of the explored metrics as functions of hyperparameters η and β (increasing $1/\eta$ or β leads to stronger regularization), for four-attractor dynamics. The red dot indicate the values used for the main analysis. (G) Clustering example for $\eta = \beta = 0.48$. (H) Clustering example for $\eta = \beta = 100$. The dashed ellipses are the two sigma contours of the Gaussian components.
