# Supplementary material for: Identification of recurrent dynamics in distributed neural populations
Source: PLoS Comput Biol. 2025 Feb 6;21(2):e1012816. doi: 10.1371/journal.pcbi.1012816 (PMC11838891; doi:10.1371/journal.pcbi.1012816)
Supplement: Attachment — Submitted filename: Response to reviewers.docx [file pcbi.1012816.s002.docx]

We are thrilled that the reviewers “agreed on the methodological relevance of the study and its potential for neuroscience” and that they “praised the level of detail of the analyses on a range of dynamical regimes.” The reviews provided constructive comments which we have carefully incorporated into the revised manuscript to further strengthen the methodology and analysis. The manuscript is improved with the revisions based on the feedback of the reviewers and we believe merits publication in this journal. Please see our itemized responses to the reviewers’ comments below:

**Reviewer #1**

Overview: This work explores the TVART dynamical systems model through a series of empirical simulations based on neuroscience models and neural data analysis. TVART, previously presented by Harris et al. (2021), models nonstationary dynamics as switching between a set of dynamics that have a low-rank tensor representation when stacked in a single 3D array. This representation enables a concise description of the dynamics with the assumptions of 1) low-rank dynamics over each window, and 2) locally static linear dynamics that switch between time windows. The primary contributions of this work is a set of experiments that explore the properties of TVART in different contexts and over different dynamical regimes. The rank, stability, and attractor dynamics are specifically studied. While this paper does present interesting analyses of the model, there are several significant weaknesses to the study. For one, the actual experiments are limited compared to the statements in the introduction and the conclusions drawn in the discussion. More significant analyses should be run to justify these statements, such as comparisons to other algorithms or studies of how to better quantify/address some of the properties shown. Furthermore, the analyses are largely circumstantial and some actually tend to suggest that the assumptions in TVART might not best match the data analyzed, reinforcing that comparisons to other methods would be valuable. Finally, as the venue is PLoS CompBio, the link with biology can be strengthened. These points and others are listed with more details below, and I think should be addressed before this work can be considered for publication.

We thank the reviewer for their insightful comments. Based on the suggestion to include more significant analyses and comparisons to algorithm, we have completed additional analyses and benchmarked performance using three alternative methods: SLDS, rSLDS and DLDS. These three alternatives were selected based on their similar assumptions of the data and in consideration of suggestions based on reviewers’ feedback. We have also amended the Discussion text to better emphasize the links of our approach to biology (see Major Point #2 below).

Major points:

1. Clarity of the core analysis: The main analysis of the work is the attractor analysis. The introduction is written such that there was an expectation of a broader analysis of the system properties that was not addressed. My recommendation to the authors is to better align the motivation with the main analysis driving the specific conclusions.

We have made the core analysis and motivation clearer in the Introduction, specifically to ensure that the introductory text better aligns with the scope of the paper. We have emphasized the main conclusions indicating the impact of prediction delay on both the error and in attractor analysis. Please see lines (87-97) in the revised manuscript.

2. Connections to Biology: The connections to biology are primarily through simulation of neurally-motivated synthetic data. It would significantly help the paper in terms of the PLoS Comp Bio community to be more explicit on what the conclusions drawn from these simulation results in terms of neural data analysis.

We have expanded the Discussion to highlight the broader implications of the results of our study. Please see the amended discussion section in the copy where modifications are highlighted in red. We copy the relevant lines (437-463) below:

“For high-dimensional neural data where the timescale of the dynamics is unknown, fitting curves to Eq (6), could provide an estimate of said timescale. Preliminary analysis of the timescales of the dynamics can help guide the choice of a time delay for prediction for a parsimonious model that considers prediction based on a single observation from the past. Being cognizant of this timescale allows for a selection of time delay that can enhance the identification of separate dynamic regimes. As indicated by the results presented here, choosing an appropriate time delay can maximize identification of attractor structure in time series.

Alternatively, autoregression can be performed based on multistep prediction error. In general, autoregressive methods, including TVART, can easily accommodate methods that include multiple time lags, although this increases the number of parameters and makes the classification problem harder. Iterating a solution over multiple steps can also be used as a strategy for predicting over longer time horizons, such an approach avoids increasing the number of parameters [32]. Nonetheless, iteration of a linear solution may not be optimal if the underlying dynamics are non-linear.

In the context of neural time series data the challenges of high-dimensional nonlinear dynamics, stochasticity and noise are also compounded by the need of having interpretable dynamics. The results presented here indicate the locally linear dynamics that can capture the temporal evolution of neural activity accurately can also reveal broader structure given a parsimonious representation of said dynamics. Such a parsimonious representation can be in terms of temporal modes of TVART or coefficients from dLDS. In any case judicious use of these methods requires more than goodness of fit approaches. Just like in the case of attractor reconstruction through time delay embedding, a choice of an appropriate time delay can help extract relevant dynamic features in time varying auto-regressive models. Moreover, analyzing error scaling as a function of prediction delay can also reveal global time scales inherent to the dynamics. ”

3. Sparsity vs. Low-rank: The TVART assumes low-rank representations of the dynamics, as emphasized in some of their experiments. The abstract, however, mentions a sparse representation, which is significantly different in terms of underlying model assumptions. Both are low dimensional but are completely different mathematically. What do the authors mean here? Is this different from the sparsity assumption mentioned in the discussion with respect to future work? Is this sparsity different from what was done in the dLDS model or the effective 1-hot sparsity in SLDS?

We appreciate this comment from the reviewer and the opportunity to clarify the issue of low-rank vs sparsity. We do assume that the tensor is low-rank and do NOT specifically assume sparsity. Thus, we have revised the abstract to avoid the word sparse in order to be more precise with our mathematical terminology. The phrase now reads, “We demonstrate that the parsimonious representation of time-varying system matrices in terms of temporal modes can recover the attractor structure of simple systems via clustering.” The matrices from TVART are indeed not sparse by construction (though they can be, depending on the data) and we hope this rephrasing helps clarify that confusion. The representation in terms of temporal coefficients from TVART is closer to the continuously varying coefficients of DLDS than to the 1-hot representation of SLDS, as the coefficients themselves are real-valued. However, the TVART coefficients are only specified once for each time window.

We simply meant to indicate, that we can compare vectors of coefficients for each time window, rather than comparing the full dynamical systems the apply to each time window separately.

4. Model Clarity: Understanding the TVART model is pretty critical to understanding the results presented. It would improve the paper a lot to have the explicit form of the model (at least the core equations) presented and described earlier for clarity. This would also help mitigate issues with variables being used that are not defined until the methods.

We thank the reviewer for this suggestion. We have provided a brief description of TVART at the beginning of the subsection “Attractor recovery benefits from increased prediction horizon”, lines 155-172. As suggested by the reviewer, this revised description includes both the core equations and concepts to provide the reader with a better understanding of the method.

5. Metrics used: Why do the authors focus on attractor convergence and not the full = trajectory. There can be many dynamical systems with similar attractors but very different paths to get there. Trajectory MSE or other metrics might help measure this.

We appreciate the reviewer’s comment and agree with this observation. We have emphasized the and expanded the results evaluated MSE and R2 values performance as a function of prediction delay (figures 7-9). In addition, we have added new analysis that includes the MSE trends from the alternative methods (SLDS, rSLDS and DLDS) to compare the error scaling for the different scenarios explored. We found that the mean square error scales exponentially with prediction delay.

6. Attractor Hopping: On lines 172-177, the authors state:

“Initially, increasing “the time delay of the prediction resulted in bigger differences among the fitted system matrices. Thus, increasing prediction delay results in clusters of the temporal mode vectors that are more clearly separated, allowing for better classification accuracy (Fig. 3H). But as the prediction delay grows too large, the fit for the system matrices degrades, consequently impacting classification accuracy”

Can this observation be due to the system hopping into another well of attraction? Including an additional analysis that narrows down the possible sources of this effect would be very helpful.

This is a very insightful comment and question. We agree that attractor hopping can be a major contributor to a decrease in classification accuracy. To address this point, we have revised the manuscript to include a discussion of attractor hoping. In particular, we describe how the average time to switch to a new attractor dramatically decreases with increasing stochastic forcing. The figure below shows the mean switching time for the two and four attractor simulations at different levels of stochastic forcing sigma:

We observe how the time to switch to a new attractor spans from thousands of steps for the low noise two attractor case, to tens of steps for the high noise four attractor case. As the reviewer suggested, hopping into a different well of attraction certainly contributes to decrease in attractor identification when the prediction horizon is larger than the average time to switch between attractors.

7. Limitations: The limitations of TVART vs other methods in light of the simulations is not sufficiently discussed in the context of the experiments. Low rank A’s seem like they might be more of a significant assumption than authors state in the discussion: “it [TVART] has minimal assumptions about the observed dynamics” as opposed to, e.g., SLDS. In the example in Fig 6, however, large jumps in R^2 for rank changes are observed, seemingly indicating that the data might have a mis-match with the low-rank assumption. Moreover, is this statement in the discussion correct?

“Alternative approaches assume the presence of a finite set of recurring system matrices [12, 17, 28] and thus may be better poised to identify transitions between different dynamics.”

From my understanding TVART also has finite dynamics that are one of a set, finite, linear combination of a smaller set of dynamics (i.e., the low-rank assumption).

We thank the reviewer for this comment. To more comprehensively address the limitations, we have made them more explicit. TVART can enforce low rank structure but does not necessarily assume it (lines 555-556). In Figure 6, the highest rank explored is actually full rank. The reviewer is correct that TVART also limits the space of possible dynamics that those that can be expressed by the combination of the spatial modes. We have clarified this limitation and emphasized that there are constraints to the possible dynamics expressed by TVART. We emphasized the distinction between methods that find an explicit finite set of discrete dynamics (e.g. SLDS, rSLDS) as an imposed constraint of the method, and methods that express the discovered dynamics as one possible combination of a given basis (where the basis can express infinitely many systems). See lines (476-487) in the discussion.

8. Other Literature & comparisons: There is minimal discussion of other models and no comparisons to the most relevant methods: SLDS/rSLDS and dLDS.

Additional literature the authors should consider is the work done in developing methods to incorporate longer time-delays into learning, [1-4], which includes work that targets locally linear models similar to TVART [4]. These models all make the same basic observation about time-scales of learning of dynamical systems (and more generally beyond one model) and offer a number of solutions.

We appreciate the suggestion and the recommended references. As discussed in our earlier responses, we have included more significant analyses and comparisons to the algorithm. We have added completed additional analyses and benchmarked performance using three alternative methods: SLDS, rSLDS and DLDS (as suggested by the reviewer). We have also included a revised discussion of the relevant literature. See lines (437-463) in the discussion.

On lines 411-412, the authors say “Thus, sparse identification of dynamics can determine whether a time-varying linear dynamical system model is appropriate for a given data set.” Is this not what dLDS already does (Mudrik et al. 2024 in the manuscript’s references).

We appreciate these observations by the reviewer. Inspired by this comment, we performed the attractor identification analysis based on dLDS as shown in the figures below (Figures 5 and 6 in the revised manuscript). As the reviewer points out, dLDS indeed has a similar capability to characterize the time-varying dynamics; below, we show how the clustering of dlDS coefficients is qualitatively similar to the clustering of TVART temporal modes. Moreover, in both cases, increasing prediction delay improves attractor identification. In the case of dLDS coefficients further improvement was achieved via temporal smoothing.

The authors say that TVART can “can find the number and occurrence of recurrent linear systems in an unsupervised manner.” This statement and others are not demonstrated in the experiments. Comparisons to SLDS on the datasets described would enable these statements to be motivated.

We thank the reviewer for this feedback. We have clarified the statement indicated above to connect it more clearly to the content of the paper. In particular, in the revised text we discuss how clustering of the temporal modes offers an approach to achieve unsupervised identification of separate recurrent linear systems. Namely, we indicate how the distinct clusters can correspond to linear approximations of the dynamics for each fixed-point attractor. This contrasts with SLDS and rSLDS where the number of subsystems is a parameter of the model.

In response to the suggestion to compare TVART with other methods, we explored SLDS, rSLDS and dLDS. Below we show how we replicated Figure 5 for these other methods.

SLDS results MSE normalized by stochastic forcing as function of prediction delay

*rSLDS results MSE normalized by stochastic forcing as function of prediction delay*

For simulations with two neural populations, both the SLDS and the rSLDS almost recapitulated the scaling behavior of TVART. The errors were closer to the upper bound determined by the eigenvalues. On the other hand, these methods did not scale well for the simulations based on human connectivity of 90 brain regions. This observation is consistent with the results presented in the original TVART paper for SLDS; namely, there was a dramatic increase in error with increasing system size.

*dLDS results MSE normalized by stochastic forcing as function of prediction delay*

The results for dLDS differed in their error scaling both between TVART and SLDS/rSLDS. For the low dimensional simulations, dLDS can capture the smooth change in locally linear operators beyond the linearization around fixed point attractors. However, for the larger scale simulations it performs similarly to TVART.

We summarize the results in the following figure included in the new version of the manuscript (figure 8):

**Minor points:**

- Fig 3G: Why is 0.9 above 1 on the y axis? If the y-axis is probability it might make sense to scale it to [0, 1] for clarity (as with Fig. 4G/H))

We appreciate the reviewer catching this fixed. We have fixed the y-axis on Figure 3. Below we include the figure with the axes appropriately labeled with a range of [0, 1].

- Line 466: approach is that it is scales

We have edited this text appropriately to: “… approach is that it scales …”

- ‘x’ is not well defined in the first experiment

We have clarified after introducing equations 1-3: “… x represents the total synaptic input for a population …”

- Line 152: forgot ‘Fig’ in the \ref

This typo has been addressed in the revised manuscript.

- This sentence might be better rephrased for clarity: “We quantify prediction accuracy by comparing the clustering results to indices representing which attractor the trajectories in a given window are closest to.”

As suggested by the reviewer, we have rephrased the indicated text for clarity. The revised text reads as follows:

“To measure how accurately the identified clusters capture attractor hoping we index each time series window by their proximity to fixed point attractors. Thus, the attractor index represents the attractor the trajectory is closest to on average for a given window. And prediction accuracy quantifies match rates between attractor and cluster indices.”

- Consider a log-scale in Figures 3H,3G to highlight the maxima

In response to this comment and others by another reviewer, we have modified Figure 3 to this below revised version with log-scale used for the x-axis.

- In Figure 7 - what is the ground truth number of clusters? Did you also run this example on synthetic Data as well?

The ground truth number of clusters is given by the number of fixed-point attractors as described in the caption for Fig. 5. We have added this information to the caption of Figure 7.

- Line 397: no need for commas

We have eliminated the commas as suggested: “The analytic and numerical results presented in this study indicate that rather than minimizing prediction error …”

**Reviewer #2**

This MS presents simulations aimed to validate TVART, an autorregressive technique to fit low-rank linear models A: X(t+delay) = A X(t) to multivariate timeseries X. The authors use a established neural-mass model to produce two synthetic, one 2-dimensional and one high-dimensional, noisy datasets, and show that TVART can infer the correct attractor dynamics with better-than-chance performance. Moreover, they show that the performance of the autorregressive fit depends non-trivially on the parameter using the delay. Intriguingly, the delay that yields the best performance is not the one yielding the lower prediction error of the fitted model. The authors outline a strategy to identify a regime of near-optimal time-delays for problems where the ground truth is unknown.

The positive outcome of TVART's validation is, in my view, not surprising, given that the original paper validates the technique using several synthetic and real data, including neural data. The non-trivial dependency of performance with the delay, on the other hand, seems new and potentially relevant for researchers that are considering using TVART to analyse high-dimensional multivariate data.

The MS is well written and the methods and results are, to the best of my knowledge, formally correct. However, I am not familiar with autoregression or the analysis of multivariate nerual data so my confidence on this review is limited. I also wonder whether the study would be of interest to the relatively broad readership of PLoS CompBio, or would rather fit better in the journal that published the TVART method (SIAM J Appl Dyn Syst).

We appreciate your thoughtful comments and suggestions. In response to your feedback and that of your fellow reviewers, we have expanded the Discussion to enhance the relevance to analysis of neural systems.

I have a few minor suggestions, but the authors should feel free to ignore them if they are not useful.

We found the comments very useful in strengthened our manuscript and discuss our response to each suggestion independently below.

- As someone not familiar with autoregressive models I had to imagine what "prediction delay" refers to when reading the subsection starting in 144. Given its central role in the results and the scope of PLoS CompBio, it might be worth to introduce the model of Eq.~4 a bit earlier (optimally, before it's used) in the Results section.

We have moved Equation 4 and the corresponding paragraph to the beginning of the subsection titled “Attractor recovery benefits from increased prediction horizon”. We also expanded that paragraph for clarification (see lines: 155-172)

- Authors state that they "matched temporal mode cluster indices to attractor indices by choosing the permutation that minimises the difference between the two" (line 163). Although I do not anticipate any methodological problem with this approach, I believe this results in a higher chance level than assumed by the authors. The accuracy in a trial would be sampled from a distribution low-truncated at the original chance level (i.e., no sample could result in an accuracy lower than 1/N_attractors); therefore, the mean would be shifted towards higher accuracies, with chance-level accuracy monotonically increasing with the variance of the single-trial accuracy. If the authors agree with my reasoning, they could account for this by either analytical computation of the mean of the truncated distribution, or by simulating the chance-level accuracy by using uniform random sampling to decide on the attractor and then choosing the most favourable permutation for each trial. This could be important to determine if lower recognition accuracies are indeed above chance levels.

This is a subtle and important point, and we thank the reviewer for identifying it. Following the reviewer’s advice we have performed simulations to recover the chance levels associated with random sampling followed by permutation. We present the resulting distributions based on the two attractor and 4 attractor simulations:

Match rate distributions based on random sampling and permutation after random sampling. (A) for the two attractor simulation and (B) for the 4 attractor simulations.

As the reviewer pointed out, the chance levels are higher than would be expected from random choice without permutation. We have included these updated estimates of the chance level in the figures in the manuscript (see below). The lowest recognition accuracies indeed approached chance levels.

- In the same vein, the authors could compute the chance level as a distributions to show up to what extend their above-chance results could have resulted from the chance distributions.

We follow the reviewer’s recommendation and have modified Figures 3 and 4 (panels G and H) to include the median chance level as well as the 5^th^ and 95^th^ percentiles (since the distributions determined through permutation are not symmetric). For convenience, we include the modified figures here, where the black lines indicate the median and the shaded region indicates the span of the 5^th^ to the 95^th^ percentile:

- Language:

161 grammatically correct, but the preposition at the end makes the sentence a bit difficult to read: consider rephrasing

We have rephrased the indicated sentence:

“We quantify prediction accuracy by comparing the clustering results to indices representing which attractor are the trajectories in a given window closest to.”

to

“To measure how accurately the identified clusters capture attractor hoping we index each time series window by their proximity to fixed point attractors. Thus, the attractor index represents the attractor the trajectory is closest to on average for a given window. And prediction accuracy quantifies match rates between attractor and cluster indices.”

192 revise the comma

We have deleted the comma.

303 missing word (probably "one")

Included missing word: “Second, fixed point attractors' coordinates may differ only in one dimension, thus appearing close in terms of their vector norm.”

**Reviewer #3**

In this paper, the authors explore the use of time-varying autoregression with low-rank tensors (TVART) to identify recurrent linear dynamics in simulated neural population data. The paper tests TVART on stochastic neural mass models with multiple stable attractors, including simple two population models and more complex models based on human brain connectivity. The authors demonstrate that TVART can recover the underlying attractor structure through the clustering of temporal modes and examine how prediction time delay impacts the ability to separate different dynamical regimes.

This paper’s detailed investigation of TVART, its properties, and its effectiveness in recovering meaningful dynamical structures is interesting and significant. However, the paper could be improved in a few areas, as described below.

Major comments and questions:

• Clarity

o Some key concepts or terms should be defined when they are first used in the text. For example, the window size M, prediction delay, and temporal delay prediction are important terms that are used in the text before it is defined. In addition, although it is defined in the Methods section what the temporal modes of TVART are, it would be helpful for the readers if the paper explains in words or visually in the main text what these temporal modes are.

We have provided a brief discussion of the method at the beginning of the subsection “Attractor recovery benefits from increased prediction horizon”. We explained the components of the TVART fit in said segment (lines 155-172).

o The paper needs more details on how the model fitting works for TVART and how the users should interpret the fitted model. For example, it is unclear how we can extract information about the attractors from the fitted TVART (Lines 147-148).

In addition to the aforementioned discussion, we explain in more detail how the attractor information can be extracted from the temporal modes. This revised description includes both the core equations and concepts to provide the reader with a better understanding of the method (lines 155-172).

• Experimental comparisons with competing methods

o The current experiments with TVART are interesting and promising, but it should be compared with competing methods. Without proper comparisons, we cannot conclude whether TVART is doing well enough in identifying recurrent dynamics in neural data. For comparisons, the authors should consider switching models such as switching linear dynamical systems, autoregressive hidden Markov models, and related variants [1; Ch. 29]. Such models have the advantage of bypassing the additional clustering step of TVART, and the number of discrete states for these switching models can be determined via cross-validation. I am curious whether such unsupervised methods can perform as well as TVART.

• Related work

o The paper should cite recent low-rank tensor-based methods [2-4] and compare and contrast TVART with them. The authors should be clear in what strengths or weaknesses TVART has compared to these similar methods.

We have added expanded the discussion section citing and comparing these methods to TVART. See lines 463-476 in the manuscript.

• Figure interpretability

o I had trouble understanding panels E and F of Figures 3 and 4. Are the red dots disjoint sets across clusters? Additional details would be helpful for the readers.

We have modified these figures and captions to improve the clarity. In particular, we changed the red dots to match the color of the corresponding clusters in subpanels C and D. With this color-coding, the dots in panels E and F correspond to the points in panels C and D but arranged in time.

• I enjoyed the level of details in Section “Derivation of MSE around a single stable fixed

point”.

Thank you! It was very nice to receive this positive comment.

• Hyperparameter selection and effect of initialization

o Lines 486-492 list the hyperparameters used for TVART. How are these hyperparameters selected? A brief discussion of how the TVART hyperparameter selection process would be beneficial.

We have added a discussion of hyperparameters and their selection in the methods section after listing the hyperparameters. Please see the amended manuscript for this discussion (lines 583-590).

o To my understanding, CP-decomposition is prone to local minima. Since TVART imposes a rank constraint via rank R CP-decomposition, I am curious whether TVART also suffers from local minima and how different initializations affect the model fits.

There seems to be indeed an impact of local minima in model fits, for instance see Figure 4 (G-H) in the manuscript. There the error bars indicate that different initializations indeed resulted in different CP-decompositions, thus leading to varying classification accuracy. However, the error scaling results indicate that these different decompositions were almost identical of the goodness of fit as measured by the mean squared error.

Minor comments:

• I would use a different word (e.g., “demonstrating”) instead of “proving” in line 53.

We have changed the wording to “demonstrating” as suggested.

• The paper is missing the word “Figure” in lines 150 and 152.

Thank you for catching this oversight! We added “Fig.” in the missing locations.

• I may have missed it, but is there a plot that demonstrates that the results are robust to the “choice of regularization method” (Line 217)? Do different colors in Figure 5 panels C and D correspond to different regularization strengths?

In Figure 5 the red line corresponds to total variation (TV) method whereas the other lines correspond to spline regularization. We did not present results for different regularization strengths. It is possible that there are values of regularization strength that may break the scaling. In response to this comment, we add the following clarification: “This result is robust to variations in fitting window width and choice of regularization method, spline or total variation, for the chosen regularization strengths”

• For panels C and D of Figures 3 and 4, I would try to decrease the size of the dots a bit.

We have decreased the size of the dots so that they are only 60% of the previous size (see plots above).
